# High BRCA1 expression is an independent prognostic biomarker in LUAD and correlates with immune infiltration

**DOI:** 10.1002/cai2.65

**Published:** 2023-04-13

**Authors:** Fengzhu Guo, Cong Li, Shuning Liu, Zhijun Li, Jingtong Zhai, Zhiwu Wang, Binghe Xu

**Affiliations:** ^1^ Department of Medical Oncology, National Cancer Center/National Clinical Research Center for Cancer/Cancer Hospital Chinese Academy of Medical Sciences and Peking Union Medical College Beijing China; ^2^ Department of Medical Oncology, Beijing Hospital, National Center of Gerontology, Institute of Geriatric Medicine Chinese Academy of Medical Sciences Beijing China; ^3^ Department of Chemoradiotherapy Tangshan People's Hospital Tangshan China

**Keywords:** bioinformatics analysis, lung adenocarcinoma, meta‐analysis, non‐small‐cell lung cancer, prognosis

## Abstract

Lung adenocarcinoma (LUAD) patients with elevated breast cancer susceptibility gene 1 (BRCA1) expression had markedly worse overall survival and progression‐free survival compared to those with reduced BRCA1 levels. In contrast, BRCA1 expression did not correlate with survival outcomes in squamous cell carcinoma patients. The overexpression of BRCA1 was an independent risk factor for LUAD and was indicative of an immune‐suppressive tumor microenvironment.

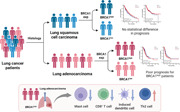

**Abbreviations**
BRCA1: breast cancer susceptibility gene 1
DEGs: differentially expressed genes
GO: Gene Ontology
iDCs: induced dendritic cells
LUAD: lung adenocarcinoma
LUSC: squamous cell carcinoma
NSCLC: non‐small‐cell lung cancer
OS: overall survival
PFS: progression‐free survival
ROC: receiver operating characteristic

Non‐small‐cell lung cancer (NSCLC) is the primary histopathological subtype of lung cancer and accounts for around 82% of all pulmonary neoplasms [1]. Breast cancer susceptibility gene 1 (*BRCA1*) is a tumor suppressor gene that regulates cellular responses to stress through DNA damage repair and is frequently mutated in breast and ovarian cancers [2]. Furthermore, previous studies have increasingly shown that the expression of *BRCA1* and the relevant poly (ADP‐ribose) polymerase inhibitors is related to the prognosis and therapeutic response of other tumors, including lung cancer [3, 4]. However, previous findings on the prognostic value of *BRCA1* for NSCLC have been inconsistent or conflicting, which may be attributed to the small number of patients from a single center or disparity in the ratio of patients with lung adenocarcinoma (LUAD) *versus* squamous cell carcinoma (LUSC) [5]. The aim of this study was to determine the prognostic value of *BRCA1* in NSCLC and its association with immune infiltration.

In this study, the clinicopathological, survival, immune infiltration, and *BRCA1* expression data of lung cancer patients retrieved from public databases and relevant articles were analyzed. The results demonstrated that *BRCA1* RNA and protein levels were significantly upregulated in LUSC and LUAD tissue compared to normal lung tissue (Figure 1a,b). Although *BRCA1* expression showed no significant correlation with the overall survival (OS) or progression‐free survival (PFS) in the overall lung cancer cohort, LUAD patients with elevated *BRCA1* expression had a markedly worse OS and PFS compared to those with reduced *BRCA1* levels. In contrast, *BRCA1* expression did not correlate with survival outcomes in LUSC patients (Figure 1c,d). The analysis of the Gene Expression Omnibus data set (GSE41271) revealed a similar association between high *BRCA1* expression and a shorter OS and PFS in LUAD (Figure 1e,f).

**Figure 1:**
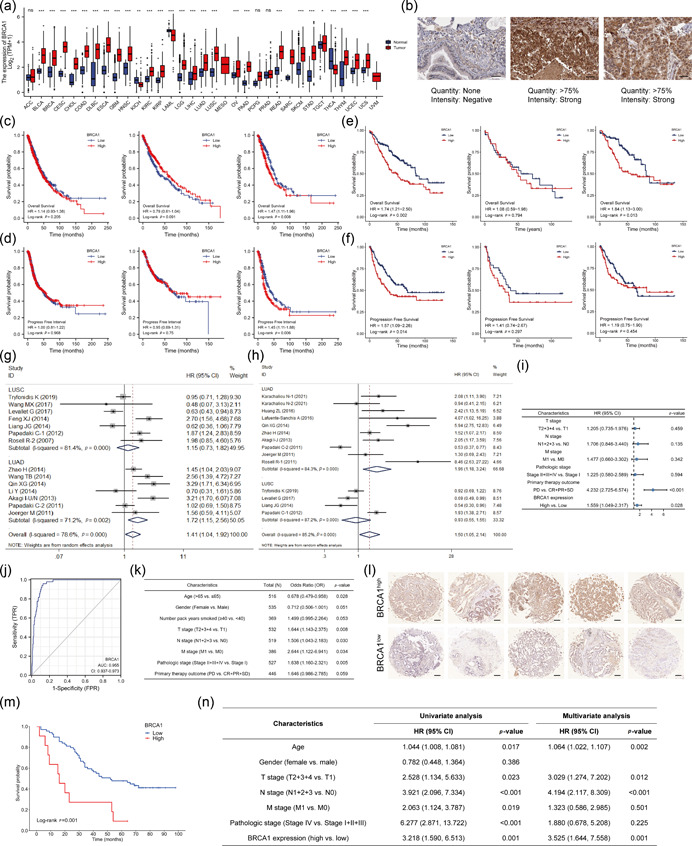
High *BRCA1* expression is an independent prognostic biomarker in lung adenocarcinoma. (a) Expression of *BRCA1* mRNA in different tumor tissue types and the corresponding normal tissues from TCGA and GTEx data sets. (b) Expression of BRCA1 protein in healthy lungs (left panel), LUSC (middle panel), and LUAD (right panel) samples from the HPA project (scale bar, 50 μm). (c, d) The OS (c) and PFS (d) of *BRCA1* ^high^ and *BRCA1* ^low^ lung cancer (left panel), LUSC (middle panel), and LUAD (right panel) patients from the TCGA database. (e, f) The OS (e) and PFS (f) of *BRCA1* ^high^ and *BRCA1* ^low^ lung cancer (left panel), LUSC (middle panel), and LUAD (right panel) patients from GEO (GSE41271). (g, h) Forest plots showing the correlation between increased BRCA1 expression and clinical prognosis in the total cohort and pathological subgroups. Results are presented as average HRs with 95% CIs of OS (g) and PFS (h) for all eligible studies. (i) Forest map showing factors influencing OS outcomes of LUAD patients as per Cox regression multivariable analysis. (j) ROC curve for the discriminatory ability of *BRCA1* using data of normal lung samples and LUAD samples from TCGA database. (k) Logistic regression analysis of the correlation between *BRCA1* expression and clinicopathological features of patients with LUAD. (l) Representative images of immunohistochemical staining for BRCA1 in LUAD tissues with high (top panel) and low (lower panel) expression of BRCA1 (scale bar, 200 μm). (m) OS of high and low BRCA1 expression in patients with LUAD as shown by Kaplan–Meier survival analysis and analyzed by a log‐rank test using the cohort data described in (l). (n) Cox univariate and multivariate regression analyses using the cohort data described in (l). *p < 0.05, **p < 0.01, ***p < 0.001. BRCA1, breast cancer susceptibility gene 1; CI, confidence interval; GEO, Gene Expression Omnibus; GTEx, Genotype‐Tissue Expression; HPA, Human Protein Atlas; HR, hazard ratio; LUAD, lung adenocarcinoma; LUSC, lung squamous cell carcinoma; OS, overall survival; PFS, progression‐free survival; ROC, receiver operating characteristic curve; TCGA, The Cancer Genome Atlas.

To further verify the abovementioned results, a systematic meta‐analysis was performed. As shown in Supporting Information: Figure S1 and Table S1, a total of 1340 potential articles were initially retrieved from PubMed, Embase, the Cochrane Library, and Web of Science data sets. The general characteristics of the 17 eligible publications involving 19 studies are shown in Supporting Information: Table S2. Data of 2765 patients, comprising 1962 males and 802 females (the gender of one patient was not given), were included in the pooled analysis. All cases were diagnosed with NSCLC, and 12 studies focused on LUAD patients, whereas 7 studies primarily included LUSC patients. The results of the meta‐analysis indicated that increased *BRCA1* portended a poor OS and PFS in patients with LUAD (Figure 1g,h). In contrast, no correlation was observed between *BRCA1* expression and prognosis in LUSC (Figure 1g,h). Due to the significant heterogeneity across the included studies, the combined hazard ratio of OS and PFS was calculated based on a random‐effects model (Figure 1g,h). Results of sensitivity analysis, meta‐regression, and publication bias are presented in supplementary materials (Supporting Information: Figures S2,3 and Tables S3,4).

Subsequently, the baseline clinicopathological information of The Cancer Genome Atlas (TCGA)‐LUAD was integrated and analyzed and exploratory analysis was conducted to fully explore its further application in clinical practice (Supporting Information: Table S5). The Cox multivariate model constructed using *BRCA1* expression and other related prognostic factors demonstrated that high *BRCA1* expression was an independent predictor for adverse OS in LUAD (Figure 1i, Supporting Information: Table S5). Moreover, the receiver operating characteristic (ROC) curve analysis suggested a robust diagnostic value of *BRCA1* in discriminating LUAD from healthy samples (Figure 1j). Univariate logistic regression further revealed that *BRCA1* overexpression was more likely in LUAD patients ≤65 years of age and with advanced tumor stage, lymph node involvement, metastasis, or advanced stage (Figure 1k). Moreover, in verifying the prognostic observation of BRCA1, the association between BRCA1 expression and OS in LUAD patients was analyzed by tissue microarray. Immunohistochemical staining indicated that high expression of BRCA1 portended a shorter OS and was an independent prognostic factor in LUAD (Supporting Information: Table S7, Figure 1l,n).

To further explore the mechanisms underlying the prognostic role of *BRCA1* in LUAD, a total of 7240 differentially expressed genes (DEGs) were screened between the *BRCA1* ^high^ and *BRCA1* ^low^ samples, of which 6555 genes were upregulated, and 685 genes were downregulated (adjusted *p* < 0.05, |Log2‐FC | > 1, Figure 2a). Gene Ontology (GO) enrichment analysis indicated that the DEGs were enriched in biological processes or cellular components related to gene expression and protein synthesis, such as mRNA trans splicing, formation of quadruple SL/U4/U5/U6 snRNP, and spliceosomal snRNP assembly (Figure 2b). Gene set enrichment analysis further revealed that more cell proliferation‐related biological processes were enriched in the *BRCA1* ^high^ group, thereby suggesting that increased *BRCA1* expression conferred a growth advantage to LUAD cells (Figure 2c). Furthermore, the expression of *BRCA1* negatively correlated with the infiltration of mast cells, CD8^+^ T cells, and induced dendritic cells (iDCs), whereas an inverse correlation was observed between *BRCA1* and Th2 cells (Figure 2d). The enrichment scores of mast cells, CD8^+^ T cells, and iDCs were significantly increased in LUAD tissues with low expression compared to those with high *BRCA1* expression, and the latter had a higher enrichment score of Th2 cells (Figure 2e).

**Figure 2:**
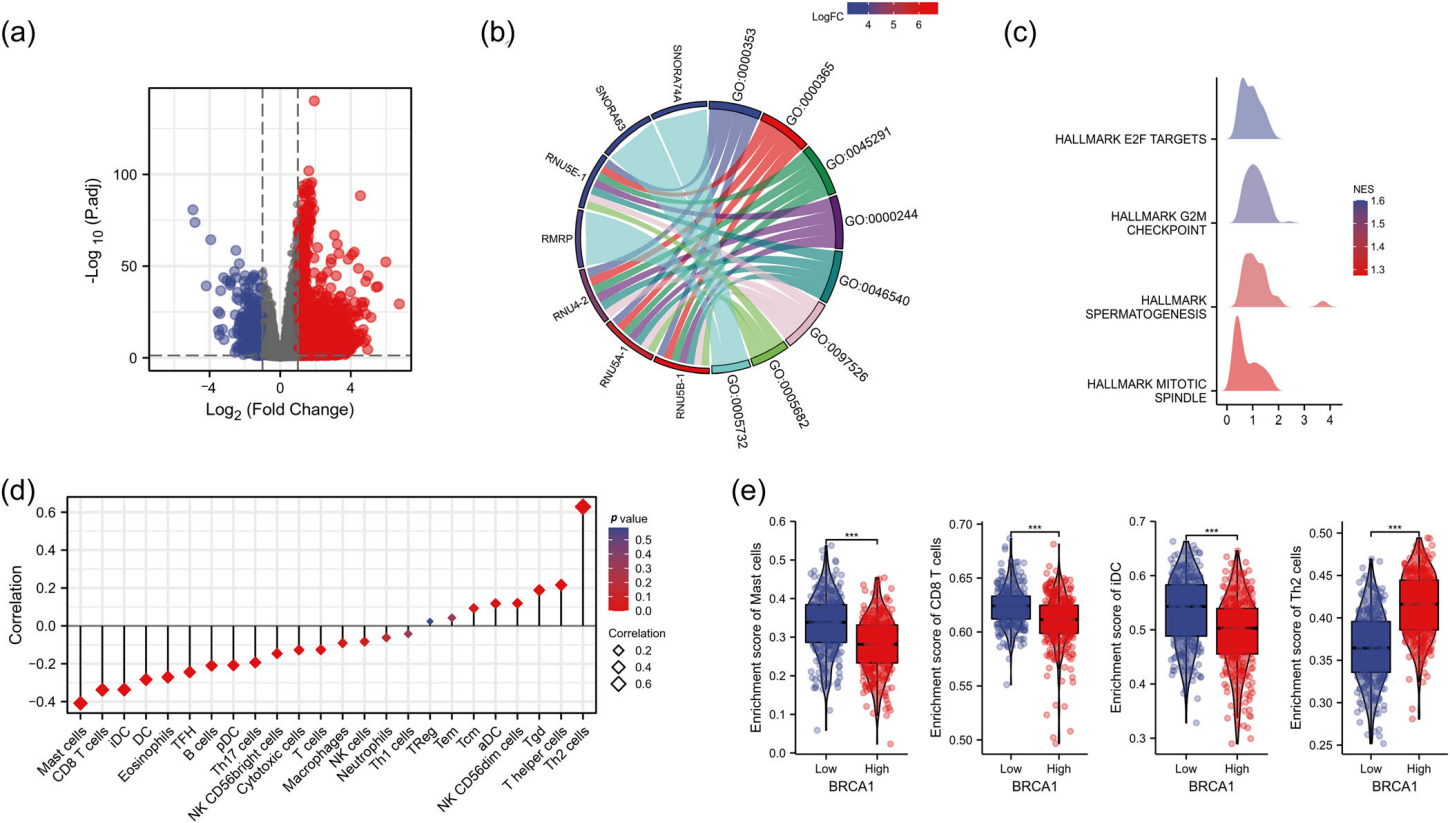
Increase in *BRCA1* expression correlates with immune infiltration in lung adenocarcinoma. (a) Volcano plot showing DEGs between *BRCA1* ^high^ and *BRCA1* ^low^ groups. (b) GO term enrichment analysis of DEGs. (c) Results of GSEA on the Hallmark gene sets archived in MSigDB. (d) Association of *BRCA1* expression with the relative abundance of different immune cell populations. The size of the diamond represents Spearman's correlation coefficient value. (e) Infiltration levels of the four most significantly different immune cells between *BRCA1* ^high^ and *BRCA1* ^low^ groups. From left to right: mast cells, CD8^+^ T cells, iDCs, and Th2 cells. ***p < 0.001. BRCA1, breast cancer susceptibility gene 1; DC, induced dendritic cell; DEG, differentially expressed gene; GESA, gene set enrichment analysis; GO, Gene Ontology; LUAD, lung adenocarcinoma; MSigDB, Molecular Signatures database. [Correction added on 20 April 2023, after first publication: The figure 2 was revised for clarity at the request of the author.]

Taken together, the overexpression of *BRCA1* is an independent risk factor for LUAD rather than LUSC, and is indicative of an immune‐suppressive tumor microenvironment. Further studies are warranted to investigate the potential role of *BRCA1* as a predictor of immunotherapy response in LUAD.

## AUTHOR CONTRIBUTIONS

**Fengzhu Guo**: Conceptualization (equal); data curation (lead); formal analysis (lead); investigation (equal); methodology (lead); project administration (supporting); software (equal); validation (lead); visualization (lead); writing—original draft (lead); writing—review and editing (supporting). **Cong Li**: Methodology (supporting); software (equal); validation (supporting). **Shuning Liu**: Data curation (supporting); formal analysis (supporting); resources (supporting); software (supporting); visualization (supporting). **Zhijun Li**: Investigation (equal); Methodology (supporting); visualization (supporting). **Jingtong Zhai**: Data curation (supporting); formal analysis (supporting); visualization (supporting). **Zhiwu Wang**: Conceptualization (equal); project administration (supporting); supervision (lead); writing—review and editing (lead). **Binghe Xu**: Conceptualization (equal); funding acquisition (lead); project administration (lead); supervision (supporting); writing—review and editing (supporting).

## CONFLICT OF INTEREST STATEMENT

Professor Binghe Xu is the member of the *Cancer Innovation* Editorial Board. To minimize bias, he was excluded from all editorial decision‐making related to the acceptance of this article for publication. The remaining authors declare no conflict of interest.

## ETHICS STATEMENT

Not applicable.

## INFORMED CONSENT

Not applicable.

## Supporting information

Supporting information.

## Data Availability

The data generated or analyzed during this study are included in this published article and its Supporting Information files; further inquiries can be directed to the corresponding author.
