# Supplementary material for: High BRCA1 expression is an independent prognostic biomarker in LUAD and correlates with immune infiltration
Source: Cancer Innov. 2023 Apr 13;2(2):91–5. doi: 10.1002/cai2.65 (PMC10686107; doi:10.1002/cai2.65)
Supplement: Supplementary file 1 — Supporting information. [file CAI2-2-91-s001.docx]

Supplementary Materials for

High BRCA1 expression is an independent prognostic biomarker in LUAD and correlates with immune infiltration

Fengzhu Guo^1,2^, Cong Li^1^, Shuning Liu^1^, Zhijun Li^1^, Jingtong Zhai^1^, Zhiwu Wang^3*^, Binghe Xu^1*^

^1^ Department of Medical Oncology, National Cancer Center/National Clinical Research Center for Cancer/Cancer Hospital, Chinese Academy of Medical Sciences and Peking Union Medical College, Beijing 100021, China

^2^ Department of Medical Oncology, Beijing Hospital, National Center of Gerontology, Institute of Geriatric Medicine, Chinese Academy of Medical Sciences, Beijing 100730, China

^3^ Department of Chemoradiotherapy, Tangshan People’s Hospital, Tangshan 063000, China

^*^Correspondence: Binghe Xu, Zhiwu Wang

Department of Medical Oncology, National Cancer Center/National Clinical Research Center for Cancer/Cancer Hospital, Chinese Academy of Medical Sciences and Peking Union Medical College, Beijing 100021, China.

Department of Chemoradiotherapy, Tangshan People’s Hospital, Tangshan 063000, China.

Email: xubingheBM@163.com, tcm2000@163.com

1. **Materials and methods**

The preferred reporting approach for systematic reviews and meta‐analyses (PRISMA) standards [1], and the epidemiology protocol for meta-analysis of observational studies were followed [2].

- 1. **Data sources**

Four investigators (FZG, CL, SNL, and ZJL) reviewed PubMed, Embase, the Cochrane Library, and Web of Science databases for relevant articles published until 30 November 2022. The studies were screened using a combination of Medical Subject Headings (MeSH) and text terms as shown in Table S1. The reference lists of each article were checked manually for additional eligible studies.

- 1. **Eligibility criteria**

Studies were selected according to the following inclusion criteria: (i) lung cancer diagnosis confirmed by pathological examination, (ii) breast cancer susceptibility gene 1 (*BRCA1*) protein expression measured by immunohistochemistry, immunofluorescence or Western blot analysis, or mRNA levels assessed via reverse transcription polymerase chain reaction, (iii) comparison of patients with high and low *BRCA1* expression, (iv) *BRCA1* correlation with at least one clinical outcome (e.g. overall survival (OS), cancer-specific survival (CSS), progression-free survival (PFS), disease-free survival (DFS) or relapse-free survival (RFS)) that can be obtained directly or estimated from the survival curves, and (v) Newcastle-Ottawa scale (NOS) score of six or more points [3]. Exclusion criteria were as follows: (i) abstracts, letters, editorials, case reports, review papers, systematic reviews or non-clinical studies, (ii) studies not published in English, (iii) studies lacking data required for analysis, and (iv) duplicate publications or repeated analyses of prior studies.

- 1. **Data extraction, endpoints, and quality assessment**

Two researchers (FZG and CL) independently screened the selected articles. Records that could not be categorized based on the title and abstract were retrieved for full-text review by two authors (SNL and ZJL). Any disagreements were resolved by discussing with a third reviewer (JTZ). The following details were extracted from each study: first author’s name, year of publication, study design, and country, patient information (number, gender, ethnicity and mean age), oncological features (predominant histology and stage), treatment approach (strategies and follow-up), clinical outcome, and hazard ratio (HR) with 95% confidence interval (CI). The primary and secondary endpoints were OS and PFS respectively. Because the CSS and OS, and DFS, RFS and PFS were similarly defined in the included studies, CSS data were included as OS, and PFS was defined as DFS or RFS for studies that did not demonstrate the PFS. The quality of each study was rated independently by two authors (ZWW and FZG) using Newcastle–Ottawa Scale (NOS) with eight items covering three aspects: selection, comparability, and outcome assessment. Articles with an NOS score of less than six points were classified as non-conforming records.

- 1. **Bioinformatics analysis**

Pan-cancer sequencing data and corresponding clinical information were downloaded from the Cancer Genome Atlas data portal (https://tcga-data.nci.nih.gov/tcga/). The separate files were merged into two matrix files of expression profiles and clinical information, and the *BRCA1*-related data was extracted. Differential expression diagrams and Kaplan-Meier curves were drawn using the R “beeswarm” and “survival” packages. The protein expression profile of *BRCA1* in lung cancer was determined using immunohistochemical data archived in the Human Protein Atlas database (http://www.proteinatlas.org). The correlation between *BRCA1* expression and clinicopathological features was analyzed using univariate logistic regression analysis. *BRCA1* expression and other prognosis-related factors were incorporated into Cox univariate and multivariate regression models in a stepwise manner using the R “survival” package to identify independent prognostic factors. The receiver operating characteristic (ROC) curve was plotted using the timeROC package to evaluate the predictive accuracy of *BRCA1*.

LUAD patients were divided into *BRCA1*^high^ and *BRCA1*^low^ groups based on *BRCA1* expression levels. The differentially expressed genes (DEGs) between the two groups were screened with the R package DESeq2 and visualized using ggplot2 (adjusted P value < 0.05, |log_2_FC| >1). Gene ontology (GO) analysis was performed with the R package GOplot and gene set enrichment analysis (GSEA) was performed using the clusterProfiler package (adjusted P value <0.05, |log_2_FC| >3). The infiltration of 24 immune cells was calculated as the relative enrichment score using the R package GSVA and compared between the two groups using the Wilcoxon rank-sum test. The results of TCGA dataset were verified on the independent Gene Expression Omnibus dataset (GSE41271) consisting of 275 patients with outcome follow-up data using R “survival” package. The expression matrix and clinical files were downloaded from the website, followed by file-merging and visualization of Kaplan-Meier curves as described previously.

- 1. **Tissue sample collection and immunohistochemistry (IHC)**

A commercial lung adenocarcinoma tissue microarray with clinicopathological features and prognostic information was purchased and used for immunohistochemical staining and evaluation of BRCA1. An anti-BRCA1 antibody was purchased from Abcam (Cat# ab16780, Cambridge, MA, USA). Briefly, the section was dewaxed in xylene and rehydrated in graded ethanol, followed by antigen retrieval, peroxidase activity quenching, and blockade. Then, the tissue section was incubated with the primary and secondary antibodies, and counterstained with hematoxylin as suggested by the manufacturer. Subsequently, the section was semiquantitatively evaluated by an experienced pathologist, and the IHC score was calculated as the percentage of positive cells × the staining intensity. Finally, patients were assigned into two arms with high and low BRCA1 expression according to the best cutoff value. Ethical approval and informed consent were obtained for all patients from the appropriate ethics committees.

- 1. **Statistical analysis**

HR was selected as the evaluation index for prognosis. Both HR and the associated 95% CI were obtained directly from the studies or were estimated based on the information reported in each trial according to the method of Parmar *et al* [4]. HR < 1 indicated favorable survival for the participants with BRCA1 overexpression. The heterogeneity of selected studies was quantified using the Cochran’s Q-test and Higgins I-squared statistic (I^2^) [5]. I^2^ > 50% or P value < 0.05 for Cochran’s Q-test indicated significant heterogeneity [5]. In case of substantial heterogeneity across studies, random-effects models were applied to calculate the pooled HR and 95% CI. Otherwise, the fixed-effects model was used [6]. Subgroup and sensitivity analyses along with meta-regression were performed to detect the potential source of heterogeneity. Publication bias was estimated statistically through Egger’s test [7, 8]. All tests were two-sided, and P value < 0.05 was considered statistically significant [9]. All meta-analyses were performed using Stata/SE version 12.0 (Stata Corporation, College Station, TX, USA), and all statistical analyses on data derived from public databases were performed using R language (v4.1.2).

1. **Sensitivity analysis, meta-regression, and publication bias for meta-analysis**

Consistent with the results of the subgroup analysis, sensitivity analysis showed that the heterogeneity and instability in the overall assessment of OS and PFS were the result of differences in tumor histological types among enrolled patients (Fig. 1g-h, Fig. S2). Meta-regression analysis was performed to detect possible sources of heterogeneity, which showed that none of the variables tested were significantly related to heterogeneity (Table S3-4). The Egger’s test indicated lack of any publication bias in the meta-analysis of OS and PFS (Fig. S3).

1. **Supplementary tables**

**Table S1** Search strategy

| Database | Search terms | | |
| --- | --- | --- | --- |
| PubMed | lung neoplasms | genes, BRCA1 | prognosis |
| Embase | pulmonary neoplasms | BRCA1 genes | outcome |
| Cochrane Library | lung neoplasm | BRCA1 gene | survival |
| Web of Science | pulmonary neoplasm | gene, BRCA1 | mortality |
|  | lung cancer | breast cancer susceptibility gene 1 | relapse |
|  | lung cancers |  | recurrence |
|  | pulmonary cancer |  |  |
|  | pulmonary cancers |  |  |
|  | cancer of the lung |  |  |
|  | cancer of lung |  |  |

**Table S2** Main characteristics of studies included in the meta-analysis

| Author | Year | Study region | Ethnicity | Study design | Sample size (M/F) | Age (years, mean or median ± SD or range) | Predominant histology | Stage | Treatment | Follow-up (months, median and range) | Outcome | HR | NOS score |
| --- | --- | --- | --- | --- | --- | --- | --- | --- | --- | --- | --- | --- | --- |
| Karachaliou N-1 [10] | 2021 | Spain | Caucasian | P | 51 (18/33) | 70 (36–85) | LUAD | IV | Targeted therapy^a^ | 45 | PFS | U | 9 |
| Karachaliou N-2 [10] | 2021 | Spain | Caucasian | P | 40 (10/30) | 65 (39–85) | LUAD | IV | Targeted therapy^b^ | 45 | PFS | U | 9 |
| Tryfonidis K [11] | 2019 | Greece | Caucasian | R | 239 (210/28)^c^ | 64 (36–87) | LUSC | I–IIIA | Mix | 96 | OS/RFS | U/M | 9 |
| Wang MX [12] | 2017 | China | Asian | R | 70 (61/9) | 61 (31–79) | LUSC | II–III | Mix | NR | OS | U/M | 9 |
| Levallet G [13] | 2017 | France | Caucasian | R | 221 (170/51) | 60 | LUSC | I–IV | Mix | 90 | OS/PFS | M | 9 |
| Lafuente-Sanchis A [14] | 2016 | Spain | Caucasian | R | 64 (54/10) | 67.8 ± 1.2 | LUAD | I | Surgery | 13 (8–36) | DFS | M | 9 |
| Feng XJ [15] | 2014 | China | Asian | P | 208 (158/50) | 64.1 (25.3–86.1) | LUSC | IIIB–IV | Chemotherapy | NR | OS | U | 8 |
| Wang TB [16] | 2014 | China | Asian | P | 366 (269/97) | 62.6 (25.5–86.4) | LUAD | IIIB–IV | Mix | NR | OS | M | 9 |
| Qin XG [17] | 2014 | China | Asian | P | 190 (130/60) | 61.5 (29–76) | LUAD | IIIB–IV | Chemotherapy | 2–60 | OS/PFS | U | 9 |
| Liang JG [18] | 2014 | China | Asian | P | 377 (269/108) | 64.6 (25.5–86.4) | LUSC | IIIB–IV | Chemotherapy | NR | OS/PFS | M | 9 |
| Zhao H [19] | 2014 | China | Asian | P | 158 (115/43) | 59 (31–79) | LUAD | IIIB–IV | Chemotherapy | NR | OS/TTP | U | 9 |
| Li Y [20] | 2014 | China | Asian | P | 34 (25/9) | 61 (40–80) | LUAD | IIIB–IV | Chemotherapy | NR | OS | U | 8 |
| Akagi I-J^d^ [21] | 2013 | US | Asian | R | 199 (97/102) | 59.4 ± 7.7 (30–76) | LUAD | I–II^e^ | Mix | NR | RFS | U | 9 |
| Akagi I-U/N^f^ [21] | 2013 | US | Caucasian/African-American | R | 92 (52/40) | 64.7 (37–90) | LUAD | I–II | None or unknown | NR | CSS | U | 9 |
| Papadaki C-1 [22] | 2012 | Greece | Caucasian | R | 100 (79/21) | 63 (34–78) | LUSC | IV | Chemotherapy | 9.9 (1.2–62.6) | OS/PFS | U/M | 9 |
| Papadaki C-2 [23] | 2011 | Greece | Caucasian | R | 131 (106/25) | 60 (37–78) | LUAD | IIIB–IV | Chemotherapy | 9.7 (1.3–84.5) | OS/PFS | U/M | 9 |
| Rosell R-1 [24] | 2011 | Spain | Caucasian | P | 54 (18/36) | 67 (22–86) | LUAD | IV | Targeted therapy | NR | PFS | M | 8 |
| Joerger M [25] | 2011 | The Netherlands | Caucasian | P | 45 (23/22) | 59.3 (45–75) | LUAD | IIIB–IV | Chemotherapy | 42 | OS/PFS | U | 9 |
| Rosell R-2 [26] | 2007 | Spain | Caucasian | R | 126 (98/28) | 64 (37–77) | LUSC | I–IIIA | Mix | 29.7 (1.7–65.9) | OS | U | 8 |

**Notes:** ^a^gefitinib; ^b^gefitinib plus olaparib; ^c^the gender information of one case was missing; ^d^Japan cohort; ^e^Two cases were unknown; ^f^the US/Norway cohort.

**Abbreviations:** SD, standard deviation; HR, hazard ratio; “U” represents univariate analysis, “M” means multivariate analysis; NOS, Newcastle–Ottawa Scale; NSCLC, non-small-cell lung cancer; LUSC, lung squamous cell carcinoma; LUAD, lung adenocarcinoma; Mix, integrative treatment including surgery and chemotherapy; OS, overall survival; RFS, relapse-free survival; PFS, progression-free survival; DFS, disease-free survival; CSS, cancer-specific survival; P, prospective; R, retrospective; NR, not reported.

**Table S3** Meta-regression analysis for overall survival

| Variable | Adjusted R^2^ | Coef (95% CI) | P value |
| --- | --- | --- | --- |
| Sample size | -10.06% | -0.03 (-0.95, 0.90) | 0.950 |
| Study design | -5.99% | 0.22 (-0.52, 0.96) | 0.532 |
| Analysis type | 13.45% | -0.51 (-1.20, 0.18) | 0.134 |
| Stage | 1.43% | -0.28 (-0.94, 0.37) | 0.364 |
| Ethnicity | -8.96% | -0.09 (-0.85, 0.66) | 0.793 |
| Histology | 4.38% | -0.40 (-1.11, 0.31) | 0.247 |
| Therapy | -7.36% | -0.11 (-0.50, 0.29) | 0.565 |

**Table S4** Meta-regression analysis for progression-free survival

| Variable | Adjusted R^2^ | Coef (95% CI) | P value |
| --- | --- | --- | --- |
| Sample size | 7.37% | 0.60 (-0.34, 1.54) | 0.192 |
| Study design | 2.33% | 0.44 (-0.50, 1.39) | 0.326 |
| Analysis type | -8.59% | -0.14 (-1.13, 0.86) | 0.766 |
| Stage | -1.35% | -0.37 (-1.21, 0.47) | 0.353 |
| Ethnicity | -4.72% | -0.28 (-1.28, 0.73) | 0.557 |
| Histology | 14.19% | -0.75 (-1.67, 0.18) | 0.105 |
| Therapy | -10.56% | -0.09 (-0.61, 0.44) | 0.728 |

**Table S5** Baseline information of LUAD patients enrolled in this study derived from the TCGA database

| Characteristics | Low expression of BRCA1 | High expression of BRCA1 |
| --- | --- | --- |
| N | 267 | 268 |
| Age, n (%) |  |  |
| ≤65 | 116 (45.5%) | 139 (54.5%) |
| >65 | 144 (55.2%) | 117 (44.8%) |
| Gender, n (%) |  |  |
| Female | 154 (53.8%) | 132 (46.2%) |
| Male | 113 (45.4%) | 136 (54.6%) |
| Number pack years smoked, n (%) |  |  |
| <40 | 101 (53.7%) | 87 (46.3%) |
| ≥40 | 79 (43.6%) | 102 (56.4%) |
| T stage, n (%) |  |  |
| T1 | 102 (58.3%) | 73 (41.7%) |
| T2 | 127 (43.9%) | 162 (56.1%) |
| T3 | 27 (55.1%) | 22 (44.9%) |
| T4 | 10 (52.6%) | 9 (47.4%) |
| N stage, n (%) |  |  |
| N0 | 184 (52.9%) | 164 (47.1%) |
| N1 | 43 (45.3%) | 52 (54.7%) |
| N2 | 30 (40.5%) | 44 (59.5%) |
| N3 | 0 (0%) | 2 (100%) |
| M stage, n (%) |  |  |
| M0 | 183 (50.7%) | 178 (49.3%) |
| M1 | 7 (28%) | 18 (72%) |
| Pathologic stage, n (%) |  |  |
| Stage I | 161 (54.8%) | 133 (45.2%) |
| Stage II | 53 (43.1%) | 70 (56.9%) |
| Stage III | 38 (45.2%) | 46 (54.8%) |
| Stage IV | 8 (30.8%) | 18 (69.2%) |
| Primary therapy outcome, n (%) |  |  |
| PD | 28 (39.4%) | 43 (60.6%) |
| SD | 20 (54.1%) | 17 (45.9%) |
| PR | 3 (50%) | 3 (50%) |
| CR | 171 (51.5%) | 161 (48.5%) |

**Abbreviations:** LUAD, lung adenocarcinoma; TCGA, The Cancer Genome Atlas; BRCA1, breast cancer susceptibility gene 1; PD, progressive disease; SD, stable disease; PR, partial response; CR, complete response.

**Table S6** Cox regression univariate analysis of factors influencing overall survival outcome of LUAD patients

| Characteristics | Total (N) | HR (95% CI) | P value |
| --- | --- | --- | --- |
| Age | 516 |  |  |
| ≤65 | 255 | Reference |  |
| >65 | 261 | 1.223 (0.916-1.635) | 0.172 |
| Gender | 526 |  |  |
| Male | 246 | Reference |  |
| Female | 280 | 0.934 (0.701-1.245) | 0.642 |
| Pack years of smoking | 363 |  |  |
| <40 | 183 | Reference |  |
| ≥40 | 180 | 1.073 (0.753-1.528) | 0.697 |
| T stage | 523 |  |  |
| T1 | 175 | Reference |  |
| T2+3+4 | 348 | 1.728 (1.229-2.431) | 0.002 |
| N stage | 510 |  |  |
| N0 | 343 | Reference |  |
| N1+2+3 | 167 | 2.601 (1.944-3.480) | <0.001 |
| M stage | 377 |  |  |
| M0 | 352 | Reference |  |
| M1 | 25 | 2.136 (1.248-3.653) | 0.006 |
| Pathologic stage | 518 |  |  |
| Stage I | 290 | Reference |  |
| Stage II+III+IV | 228 | 2.933 (2.173-3.958) | <0.001 |
| Primary therapy outcome | 439 |  |  |
| CR+PR+SD | 368 | Reference |  |
| PD | 71 | 3.618 (2.539-5.155) | <0.001 |
| *BRCA1* expression | 526 |  |  |
| Low | 265 | Reference |  |
| High | 261 | 1.476 (1.106-1.969) | 0.008 |

**Abbreviations:** LUAD, lung adenocarcinoma; HR, hazard ratio; CR, complete response; PR, partial response; SD, stable disease; PD, progressive disease; BRCA1, breast cancer susceptibility gene 1.

**Table S7** Baseline information of LUAD patients included in the tissue microarray

| Characteristics | Low expression of BRCA1 | | High expression of BRCA1 |
| --- | --- | --- | --- |
| N | 69 | 11 | |
| Age, mean ± SD | 59.14 ± 9.12 | 59.64 ± 9.98 | |
| Gender, n (%) |  |  | |
| Female | 32 (46.38%) | 7 (63.64%) | |
| Male | 37 (53.62%) | 4 (36.36%) | |
| T stage, n (%) |  |  | |
| T1 | 16 (23.19%) | 1 (9.09%) | |
| T2 | 29 (42.03%) | 3 (27.27%) | |
| T3 | 18 (26.09%) | 3 (27.27%) | |
| T4 | 6 (8.70%) | 4 (36.36%) | |
| N stage, n (%) |  |  | |
| N0 | 34 (49.28%) | 3 (27.27%) | |
| N1 | 15 (21.74%) | 2 (18.18%) | |
| N2 | 19 (27.54%) | 5 (45.45%) | |
| N3 | 1 (1.45%) | 1 (9.09%) | |
| M stage, n (%) |  |  | |
| M0 | 52 (75.36%) | 8 (72.73%) | |
| M1 | 17 (24.64%) | 3 (27.27%) | |
| Pathologic stage, n (%) |  |  | |
| Stage I | 14 (20.29%) | 0 (0.00%) | |
| Stage II | 30 (43.48%) | 4 (36.36%) | |
| Stage III | 19 (27.54%) | 4 (36.36%) | |
| Stage IV | 6 (8.70%) | 3 (27.27%) | |

**Abbreviations:** LUAD, lung adenocarcinoma; BRCA1, breast cancer susceptibility gene 1; SD, standard deviation.

1. **Supplementary figures**

**
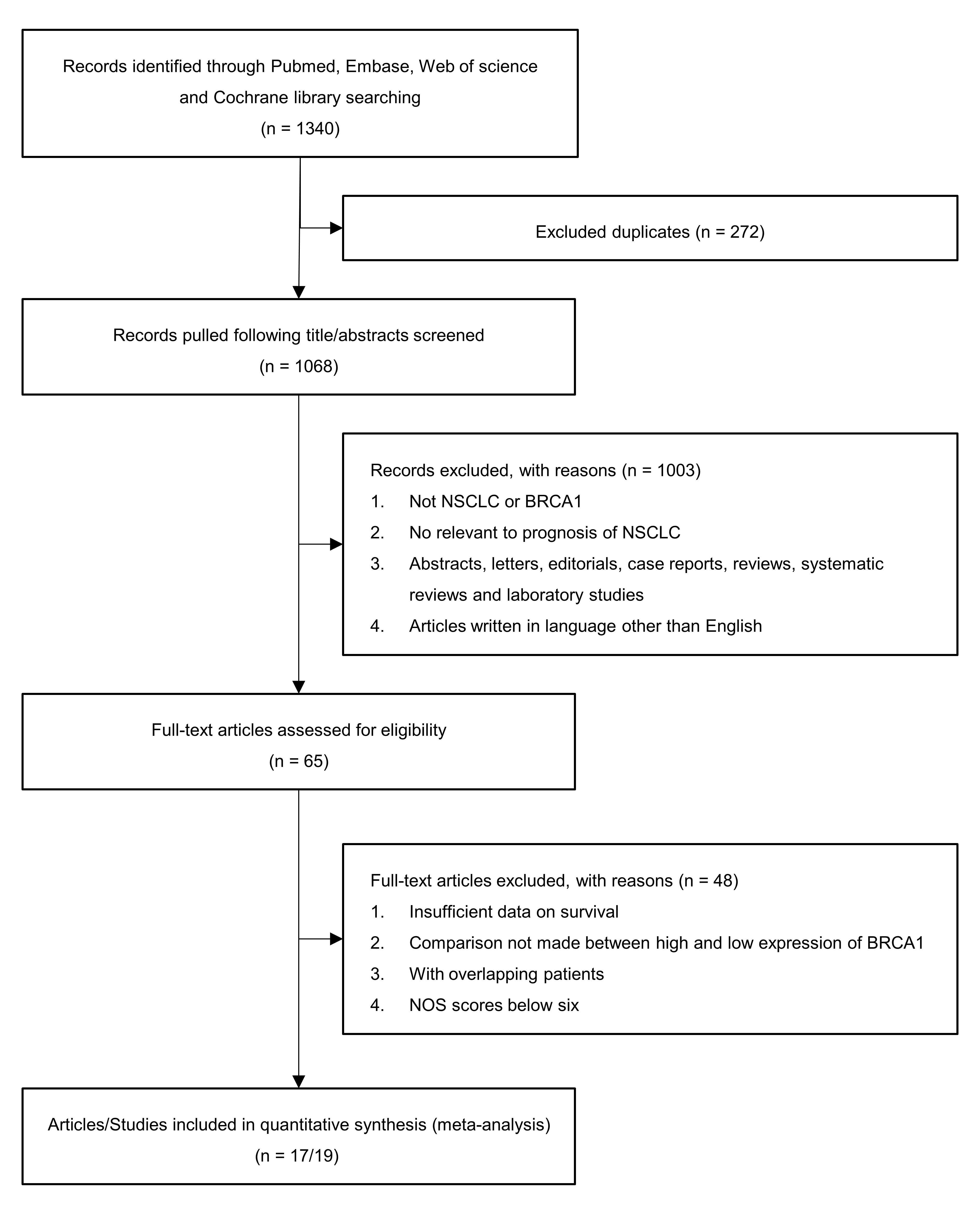
**

**Fig. S1** Flow diagram for selection of eligible studies.

**Abbreviations:** NSCLC, non-small-cell lung cancer; BRCA1, breast cancer susceptibility gene 1; NOS, Newcastle-Ottawa Scale.

**
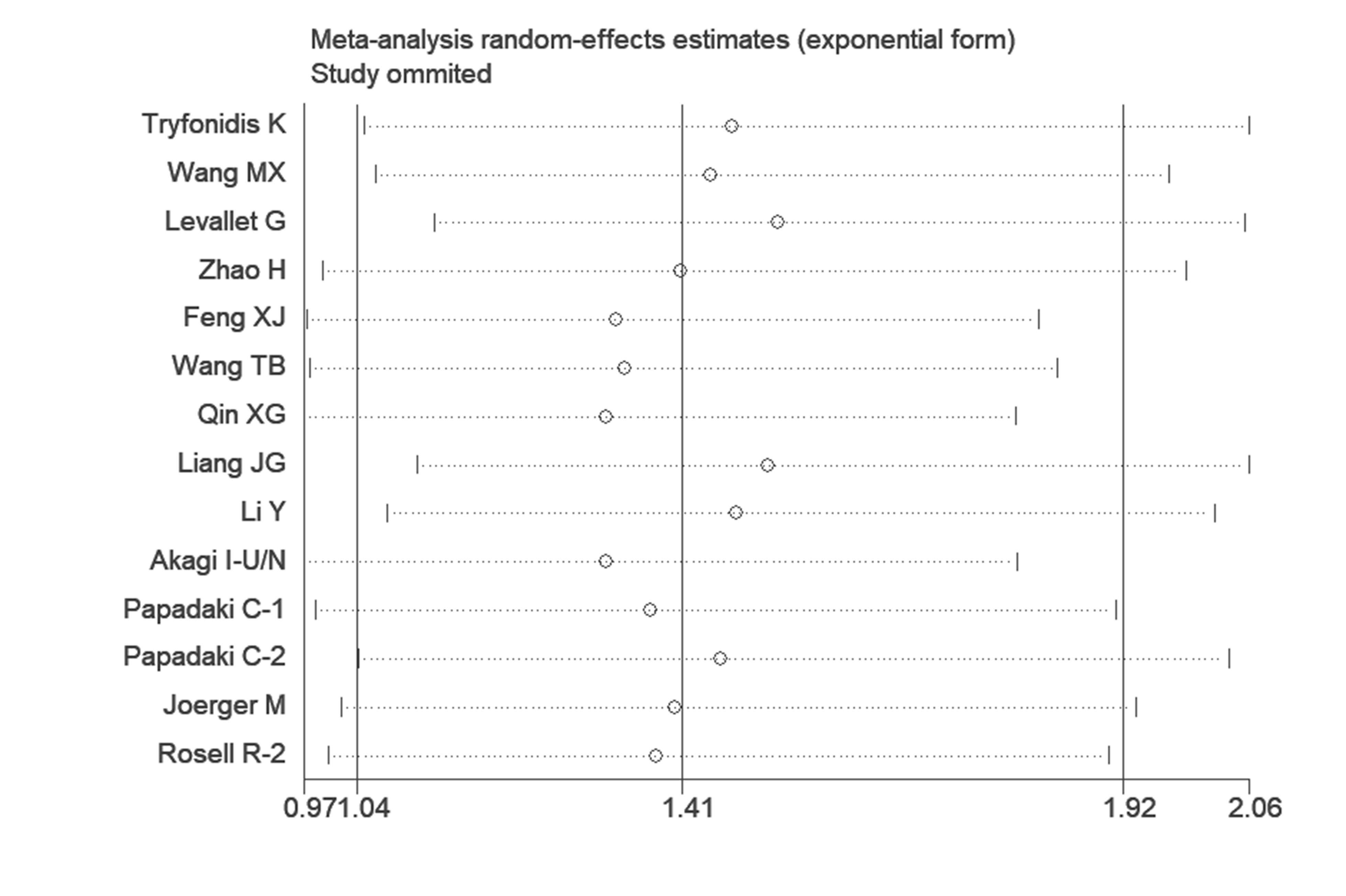
**

**
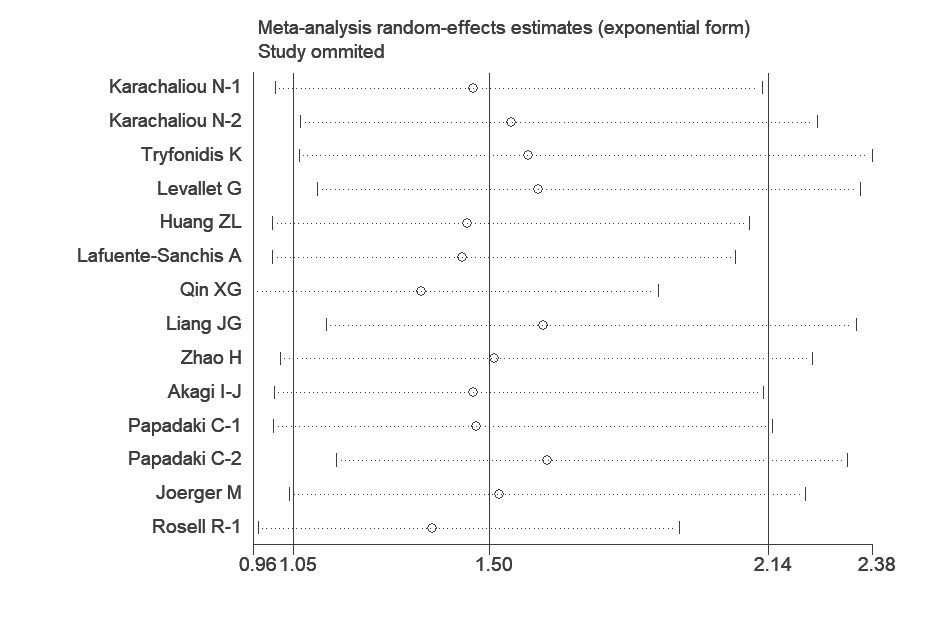
**

**Fig. S2** Sensitivity analysis to detect potential sources of heterogeneity for pooled OS (top panel) and PFS (lower panel).

**Abbreviations:** OS, overall survival; PFS, progression-free survival.

**
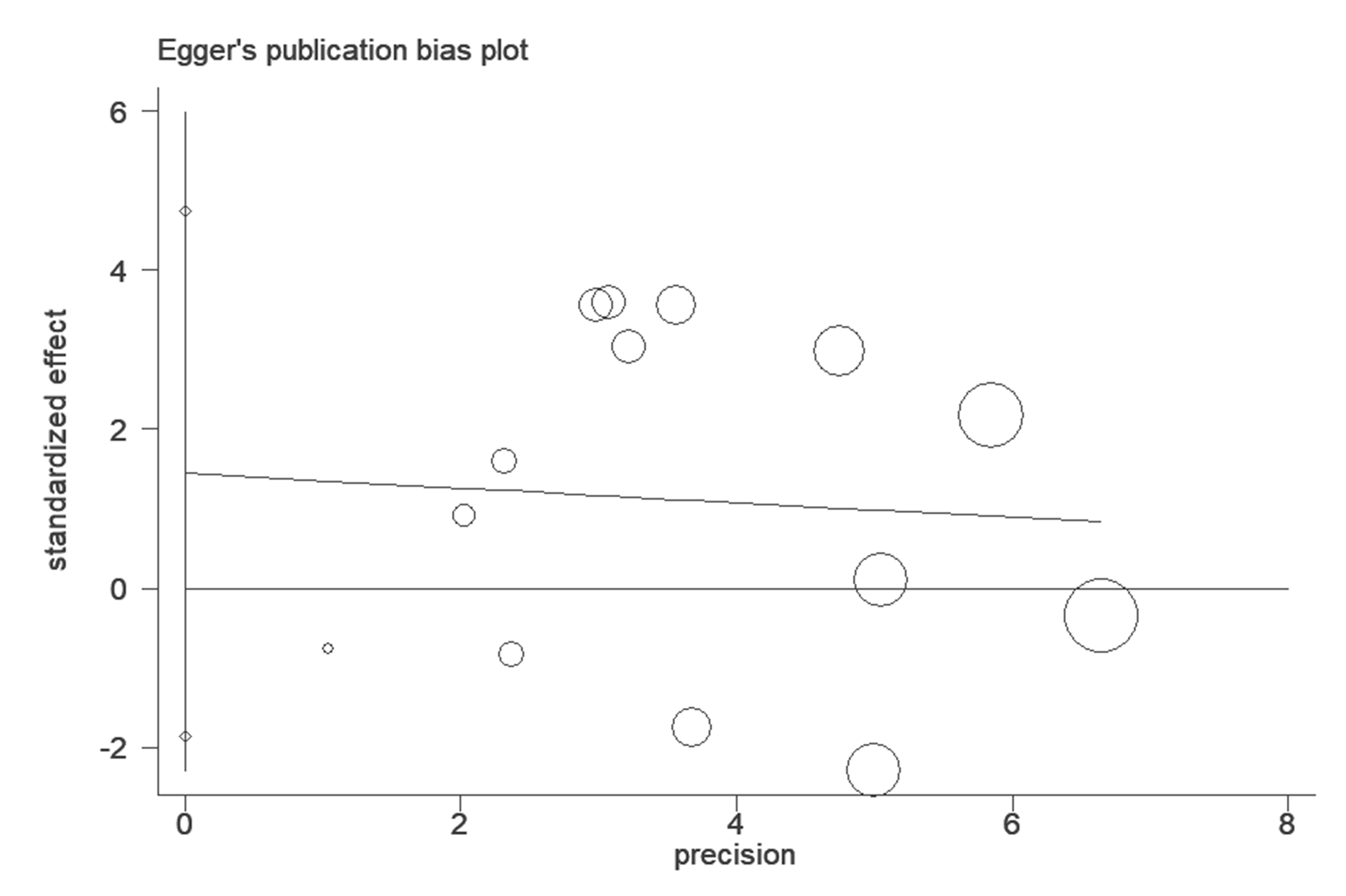
**

**
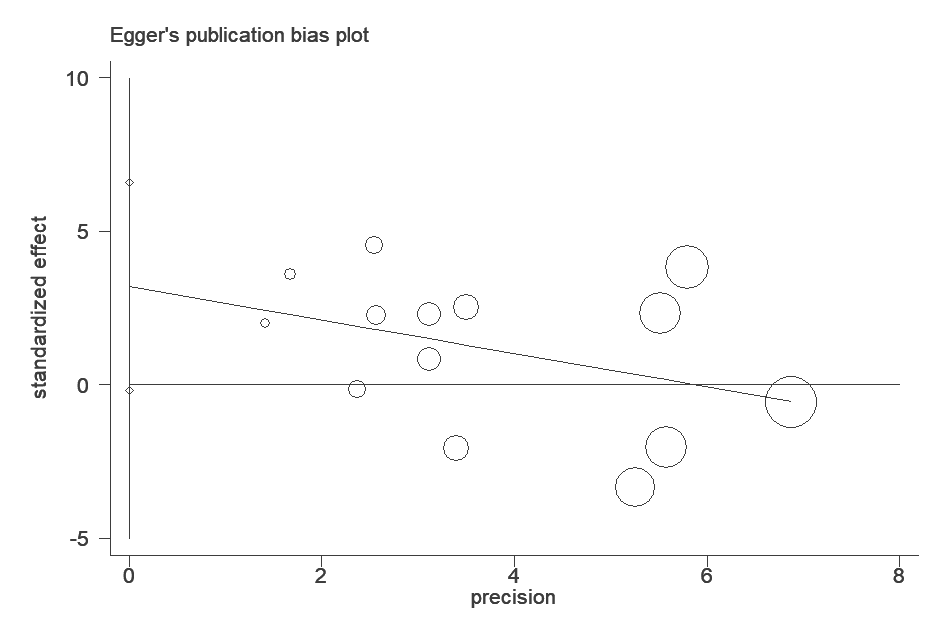
**

**Fig. S3** Egger’s test results showing publication bias for combined OS (top panel) and PFS (lower panel).

**Abbreviations:** OS, overall survival; PFS, progression-free survival.

**References**

1. Moher D, Liberati A, Tetzlaff J, Altman DG: Preferred reporting items for systematic reviews and meta-analyses: the PRISMA statement. Bmj. 2009;339:b2535.

2. Stroup DF, Berlin JA, Morton SC, Olkin I, Williamson GD, Rennie D, Moher D, Becker BJ, Sipe TA, Thacker SB: Meta-analysis of observational studies in epidemiology: a proposal for reporting. Meta-analysis Of Observational Studies in Epidemiology (MOOSE) group. Jama. 2000;283:2008-2012.

3. Le May MR, Singh K, Wells GA: Efficacy of Radial Versus Femoral Access in the Acute Coronary Syndrome: Is it the Operator or the Operation That Matters? JACC Cardiovasc Interv. 2015;8:1405-1409.

4. Parmar MK, Torri V, Stewart L: Extracting summary statistics to perform meta-analyses of the published literature for survival endpoints. Stat Med. 1998;17:2815-2834.

5. Higgins JP, Thompson SG, Deeks JJ, Altman DG: Measuring inconsistency in meta-analyses. Bmj. 2003;327:557-560.

6. Zintzaras E, Ioannidis JP: Heterogeneity testing in meta-analysis of genome searches. Genet Epidemiol. 2005;28:123-137.

7. Egger M, Davey Smith G, Schneider M, Minder C: Bias in meta-analysis detected by a simple, graphical test. Bmj. 1997;315:629-634.

8. Begg CB, Mazumdar M: Operating characteristics of a rank correlation test for publication bias. Biometrics. 1994;50:1088-1101.

9. Duval S, Tweedie R: Trim and fill: A simple funnel-plot-based method of testing and adjusting for publication bias in meta-analysis. Biometrics. 2000;56:455-463.

10. Karachaliou N, Arrieta O, Giménez-Capitán A, Aldeguer E, Drozdowskyj A, Chaib I, Reguart N, Garcia-Campelo R, Chen JH, Molina-Vila MA, Rosell R: BRCA1 Expression and Outcome in Patients With EGFR-Mutant NSCLC Treated With Gefitinib Alone or in Combination With Olaparib. JTO Clin Res Rep. 2021;2:100113.

11. Tryfonidis K, Papadaki C, Assele S, Lagoudaki E, Menis J, Koutsopoulos A, Trypaki M, Tsakalaki E, Sfakianaki M, Hasan B, et al: Association of BRCA1, ERCC1, RAP80, PKM2, RRM1, RRM2, TS, TSP1, and TXR1 mRNA expression levels between primary tumors and infiltrated regional lymph nodes in patients with resectable non-small cell lung cancer. Pharmacogenomics Journal. 2019;19:15-24.

12. Wang M, Li W, Xing X, Zhang D, Lei J, Li G: BRCA1 and STMN1 as prognostic markers in NSCLCs who received cisplatin-based adjuvant chemotherapy. Oncotarget. 2017;8:80869-80877.

13. Levallet G, Dubois F, Fouret P, Antoine M, Brosseau S, Bergot E, Beau-Faller M, Gounant V, Brambilla E, Debieuvre D, et al: MSH2/BRCA1 expression as a DNA-repair signature predicting survival in early-stage lung cancer patients from the IFCT-0002 Phase 3 Trial. Oncotarget. 2017;8:4313-4329.

14. Lafuente-Sanchis A, Zuniga A, Galbis JM, Cremades A, Estors M, Martinez-Hernandez NJ, Carretero J: Prognostic value of ERCC1, RRM1, BRCA1 and SETDB1 in early stage of non-small cell lung cancer. Clin Transl Oncol. 2016;18:798-804.

15. Feng X-j, Qin X-g, Zang L, Feng H, Wang W-l, Liu D, Li P-f: ERCC1 and BRCA1 mRNA expression predicts the clinical outcome of non-small cell lung cancer receiving platinum-based chemotherapy. Pakistan Journal of Medical Sciences. 2014;30:488-492.

16. Wang TB, Zhang NL, Wang SH, Li HY, Chen SW, Zheng YG: Expression of ERCC1 and BRCA1 predict the clinical outcome of non-small cell lung cancer in patients receiving platinum-based chemotherapy. Genet Mol Res. 2014;13:3704-3710.

17. Qin X, Yao W, Li W, Feng X, Huo X, Yang S, Zhao H, Gu X: ERCC1 and BRCA1 mRNA expressions are associated with clinical outcome of non-small cell lung cancer treated with platinum-based chemotherapy. Tumor Biology. 2014;35:4697-4704.

18. Liang JG, Jin ZY, Gao XD, Te MR, Ge LH, Wang CL: Predictive role of RRM1 and BRCA1 mRNA expression on the clinical outcome of advanced non-small cell lung cancer. Genet Mol Res. 2014;13:5292-5298.

19. Zhao H, Zhang H, Du Y, Gu X: 【high & low】5. Prognostic significance of BRCA1, ERCC1, RRM1, and RRM2 in patients with advanced non-small cell lung cancer receiving chemotherapy. Tumour Biol. 2014;35:12679-12688.

20. Li Y, Wang LR, Chen J, Lou Y, Zhang GB: First-line gemcitabine plus cisplatin in nonsmall cell lung cancer patients. Dis Markers. 2014;2014:960458.

21. Akagi I, Okayama H, Schetter AJ, Robles AI, Kohno T, Bowman ED, Kazandjian D, Welsh JA, Oue N, Saito M, et al: Combination of protein coding and noncoding gene expression as a robust prognostic classifier in stage I lung adenocarcinoma. Cancer Res. 2013;73:3821-3832.

22. Papadaki C, Sfakianaki M, Ioannidis G, Lagoudaki E, Trypaki M, Tryfonidis K, Mavroudis D, Stathopoulos E, Georgoulias V, Souglakos J: ERCC1 and BRAC1 mRNA expression levels in the primary tumor could predict the effectiveness of the second-line cisplatin-based chemotherapy in pretreated patients with metastatic non-small cell lung cancer. J Thorac Oncol. 2012;7:663-671.

23. Papadaki C, Tsaroucha E, Kaklamanis L, Lagoudaki E, Trypaki M, Tryfonidis K, Mavroudis D, Stathopoulos E, Georgoulias V, Souglakos J: Correlation of BRCA1, TXR1 and TSP1 mRNA expression with treatment outcome to docetaxel-based first-line chemotherapy in patients with advanced/metastatic non-small-cell lung cancer. Br J Cancer. 2011;104:316-323.

24. Rosell R, Molina MA, Costa C, Simonetti S, Gimenez-Capitan A, Bertran-Alamillo J, Mayo C, Moran T, Mendez P, Cardenal F, et al: Pretreatment EGFR T790M mutation and BRCA1 mRNA expression in erlotinib-treated advanced non-small-cell lung cancer patients with EGFR mutations. Clin Cancer Res. 2011;17:1160-1168.

25. Joerger M, deJong D, Burylo A, Burgers JA, Baas P, Huitema ADR, Beijnen JH, Schellens JHM: Tubuline, BRCA1, ERCC1, Abraxas, RAP80 mRNA expression, p53/p21 immunohistochemistry and clinical outcome in patients with advanced non small-cell lung cancer receiving first-line platinum-gemcitabine chemotherapy. Lung Cancer. 2011;74:310-317.

26. Rosell R, Skrzypski M, Jassem E, Taron M, Bartolucci R, Sanchez JJ, Mendez P, Chaib I, Perez-Roca L, Szymanowska A, et al: BRCA1: a novel prognostic factor in resected non-small-cell lung cancer. PLoS One. 2007;2:e1129.
